# Analysis of Adherence to Thromboprophylaxis and Incidence of Venous Thromboembolism After Lower Limb Orthopaedic Surgery

**DOI:** 10.7759/cureus.19746

**Published:** 2021-11-19

**Authors:** Miles W Benjamin, Abeku Koomson, Hany Ismaiel

**Affiliations:** 1 Trauma and Orthopaedics, Barnsley Hospital NHS Foundation Trust, London, GBR; 2 Orthopaedics, Imperial College London, London, GBR; 3 Trauma and Orthopaedics, Barnsley Hospital NHS Foundation Trust, Barnsley, GBR

**Keywords:** audit, pharmacological prophylaxis, mechanical prophylaxis, surgery, orthopaedics, venous thromboembolism

## Abstract

Background

The economic burden caused by venous thromboembolism (VTE) to the National Health Service (NHS) is approximately £640 million. There is a significant national drive for VTE prophylaxis prescription given the high morbidity and mortality rates associated with VTE following lower limb orthopaedic surgery. The primary objective of this analysis was to examine the adherence to the updated VTE guidelines, NG89, by the National Institute for Health and Care Excellence (NICE) regarding prophylaxis for patients undergoing lower limb orthopaedic surgery, where the weight-bearing status will be reduced postoperatively, in an urban community hospital.

Methodology

We looked at 586 patients who underwent elective lower limb orthopaedic surgeries over a two year time period. We reviewed their VTE prophylaxis administration. Results were shared with the Hospital Thrombosis Committee department. Education was provided to the relevant staff and hospital policy for VTE prophylaxis. The primary endpoint was to compare the proportion of patients receiving prophylaxis as per the hospital guidelines as well as complications arising from VTE.

Results

A total of 586 patients were included in this audit. Compliance with VTE pharmacological prophylaxis was recorded, as well as weight-bearing status advised postoperatively. Compliance with prophylaxis in patients who were non-weight-bearing postoperatively was 54.8%. There were three cases of recorded VTE; however, in all cases, appropriate VTE prophylaxis has been prescribed.

Conclusion

Increasing hospital-wide awareness and education of VTE and the fatal complications is imperative. All patients should be administered VTE prophylaxis as an inpatient and on discharge if their weight-bearing status is affected following lower limb orthopaedic surgery. Although our compliance rate for prescription of VTE prophylaxis did not achieve the standards set by NICE, all cases of recorded VTE had been correctly prescribed VTE prophylaxis on discharge.

## Introduction

Venous thromboembolism (VTE), which includes deep vein thrombosis (DVT) and pulmonary embolism (PE), is a common complication during and after hospitalisation, which can be fatal. The estimated number of DVT and PE events in the EU is 104-183 per 100,000 person-years [1]. Hospital-acquired VTE covers all VTE that occurs in the hospital and within 90 days after hospital admission. Without prophylaxis, PE is responsible for 5% to 10% of deaths in hospitalised patients [2]. The cost of treating VTE and related morbidity is substantial; estimates of the combined direct and indirect costs are approximately £640 million [3]. Some patients may not receive appropriate thromboprophylaxis due to risk factors that indicate they are at a high risk of bleeding. Many patients continue to be at risk of VTE after discharge; therefore, post-discharge prophylaxis is an important component of treatment [4]. Although national and international thromboprophylaxis guidelines have repeatedly recommended thromboprophylaxis of patients admitted to hospital, only 40% to 50% of medical patients and 60% to 75% of surgical patients receive adequate thromboprophylaxis [5-7]. 

Patients who undergo orthopaedic surgery are at a greater risk of VTE complications and represent a population where an undebated indication for pharmacological thromboprophylaxis is needed [4,8]. Therefore, in patients undergoing orthopaedic surgery and those with orthopaedic trauma where mobility is limited, VTE prophylaxis and adherence to the respective guidelines is paramount [3]. A recent Cochrane review reported a range of 4.3% and 40% incidence of VTE following lower limb immobilisation [9]. Virchow's Triad describes factors, which contribute to thrombosis: stasis of blood flow, endothelial injury, and hypercoagulability [10]. All three factors are present in patients who are candidates for orthopaedic surgery due to pre-operative or post-operative immobilisation and protected weight-bearing.

According to the National Institute for Health and Care Excellence (NICE) guidelines (NG89), VTE risk factors include age, obesity, cancer, thrombophilia, family history of VTE, operation time, anaesthesia, pregnancy, and immobility [7]. NICE guidelines suggest prophylaxis with low molecular weight heparin (LMWH) or fondaparinux for patients with lower limb immobilisation whose risk of VTE outweighs the risk of bleeding [7].

Pharmacological thromboprophylaxis has been shown to be effective and safe in both medical and surgical patient populations [11]. Thromboprophylaxis with unfractionated heparin (UFH), LMWH, or fondaparinux in surgical settings diminishes the rate of symptomatic and asymptomatic VTE by at least 60% compared with placebo [12,13]. LMWH has been found to be superior to both UFH and warfarin for the prevention of a DVT and results in significantly fewer minor bleeding complications when compared to UFH, but significantly more minor bleeding when compared to warfarin [14]. A Cochrane review of eight randomised control trials found a 60% risk reduction in symptomatic DVT in patients with lower leg immobility with daily prophylaxis with LMWH compared to those without prophylaxis or with placebo [9].

Adherence to guidelines is higher in surgical patients but remains limited [5,6]. The primary objective of this retrospective analysis was to examine the adherence to the updated VTE guidelines, NG89, by NICE regarding prophylaxis for patients undergoing lower limb orthopaedic surgery where the weight-bearing status will be reduced postoperatively, in an urban community hospital. Additional aims of the analysis were to determine the incidence of PE following lower limb surgery in non-weight-bearing patients and to determine if there is evidence of complications of PE in those patients discharged without prophylaxis.

## Materials and methods

Study design

The population included all patients who underwent elective lower limb surgery between July 2016 and September 2018 at Barnsley Hospital, United Kingdom. The inclusion criteria for the audit were patients who underwent lower limb surgery and completed at least 90 days of follow-up postoperatively. The exclusion criteria were patients who underwent elective total hip replacements or total knee replacements, as there was already an implemented protocol for these patients. We excluded patients whose data we were unable to obtain electronically. The outcome studied was hospital-acquired VTE in the postoperative period of 90 days as defined by NICE [7]. The Barnsley District General Hospital operating theatre electronic database was used to identify patients. All patients underwent a risk assessment pre-operatively using the VTE risk assessment tool based on NICE guidelines [7]. Patients underwent follow-up reviews after surgery. During the clinic consultation, patients were reviewed in the fracture clinic by either a surgical registrar or consultant. If the clinician had a suspicion of a VTE event, the patient was referred for appropriate imaging and started on treatment dose anticoagulants in line with local hospital protocol. Patients were determined to have a VTE event according to the radiology report. Those patients who presented with suspected DVT were sent for ultrasound doppler scanning. Those who presented with symptoms suspicious of PE were sent for computed tomography pulmonary angiography.

Variables

Patient characteristics (age, gender), admission date, discharge date, preoperative diagnosis, operative procedure, VTE risk assessment completion, VTE risk factors, anticoagulant on discharge, weight-bearing status and VTE complication details were collected. Surgical registrar grade or consultants performed all operations.

Outcomes and definitions

NICE guidelines state that VTE occurring up to 90 days from surgery is classed as a hospital-related VTE [7]. Lower limb immobilisation is defined as: “Any clinical decision taken to manage the affected limb in a way that would prevent normal weight-bearing status and/or use of that limb” [7]. This was the definition used in the study.

The primary outcome was the rate of symptomatic VTE within 90 days of surgery. Secondary outcomes reviewed in those patients with VTE were types and length of prophylaxis treatment, prophylaxis type according to each surgery type, time from surgery to VTE and all‐cause mortality within three months.

Data collection and statistical methods

Data was collected from hospital electronic records. A data collection tool was created and information transferred to a database. In order to reduce the chance of data entry errors, a ‘data validation’ function was used where possible. All data collected by one observer were independently checked by a second observer for inaccuracies. All statistical analysis was performed using SPSS v.25.0 (IBM Corp., Armonk, New York).

## Results

Data validation

A total of 586 data items (joints) were cross-checked by a second observer, 30 needed corrections.

Population demographics

**Figure 1:**
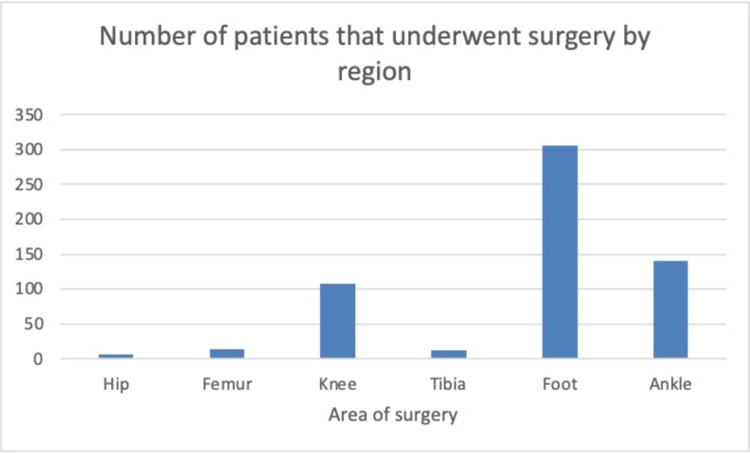
Number of patients that underwent surgery by region

In our patient population, males represented 41.1% (n = 241) and females 58.9% (n = 345), with a mean age of 51 years. Figure 1 demonstrates the number of operations by the lower limb region. Among the patients included in the study, six underwent hip surgery, 14 underwent femur surgery, 108 underwent knee surgery, 12 underwent tibia surgery, 140 underwent ankle surgery, and 306 underwent foot surgery.

Weight-bearing status & discharge anticoagulation

The weight-bearing status stated in the post-operative instructions was recorded for each patient. Three hundred seventy-six patients (64.2%) were full weight-bearing (FWB), 210 (35.8%) were not FWB. We then looked at the anticoagulation each patient had been prescribed on discharge by weight-bearing status. Of the patients with non/partial-weight-bearing status, 115 (54.8%) were prescribed an anticoagulant on discharge.

Anticoagulation

402/586 (68.7%) of patients were not prescribed pharmacological VTE prophylaxis on discharge. Within this group, 94/402 (23.3%) were not fully weight-bearing postoperatively. No cases of VTE were identified within this group. 

VTE outcomes

VTE outcomes were looked at in patients via clinic letters up to 90 days following surgery. Of the 586 patients looked at, three were found to have developed VTE within 90 days of surgery. These three patients had been prescribed six weeks of LMWH on discharge. All three cases were DVT, with no PE identified. 

VTE Cases

There were three cases of DVT identified in our study.

*Case One* This patient had an operation on their foot, which postoperatively required a partial weight-bearing status. The patient was administered LMWH as an inpatient and, on discharge, was given six weeks worth of LMWH. The patient presented one week after discharge with right leg swelling. Doppler ultrasound confirmed a thrombus in the peroneal vein of the affected limb.

*Case Two* This patient had an operation on their ankle, which postoperatively required a non-weight-bearing status. The patient was administered LMWH as an inpatient and, on discharge, was given six weeks worth of LMWH. The patient presented four weeks after discharge with left leg swelling. Doppler ultrasound confirmed a thrombus in the distal iliac vein, common femoral vein, femoral vein, and popliteal vein.

*Case Three* This patient underwent an operation on their ankle, which postoperatively required a non-weight-bearing status. The patient was administered LMWH as an inpatient and, on discharge, was given six weeks worth of LMWH. The six-week course of VTE prophylaxis was completed. The patient presented two months after discharge with left leg swelling. Doppler ultrasound confirmed a thrombus in the left femoral vein.

## Discussion

In our audit, only three out of 586 (0.5%) patients had a VTE event, and all of these were DVT. In each of the three cases, patients had received inpatient pharmaceutical VTE prophylaxis. In addition, they had been prescribed the appropriate anticoagulant on discharge from the hospital. Bala et al. looked at the contribution of patients' own comorbidities to the risk of VTE following lower limb surgery. They concluded that not all VTEs can be attributed to surgery and demonstrated that nearly 50% of DVTs may be related to the baseline health of the patients [15]. This is an important consideration within the context of analysing the cause of the VTEs. Therefore, further analysis on the baseline characteristics of the patients should take place as well as consideration of how often these patients with 'at-risk' characteristics should be followed up. The low incidence of VTE highlights the importance of continuing to advocate the use of LMWH as a form of VTE prophylaxis. Without prophylaxis, the incidence of VTE can be as high as 88% [5]. In this cohort, LMWH has been shown to save lives without any complications of over anticoagulation.

54.8% of our patients who were deemed at higher risk of developing VTE related complications were prescribed appropriate medication on discharge. This study echoes the results of a similar study conducted. This result is also comparable to the audit of Patel et al. that looked at the number of patients undergoing elective colorectal cancer surgery who were prescribed adequate VTE medication on discharge. Their first audit cycle revealed that VTE standards were not met as only 44% of patients were prescribed VTE medication on discharge [16]. 

According to NICE guidelines, all patients should have VTE risk assessments performed [7]. At our hospital trust, the risk assessments had initially been performed using written documentation methods, but this was improved with the computer-based clinical monitoring system VitalPAC. This system can alert us when compliance levels are not met.

Compliance is a difficult measure to record once a patient has been discharged home. LMWH requires the patient to administer an injection into the abdominal subcutaneous fat. This can be a painful process, and thus patients may be reluctant. Aspirin may be an effective, safe and inexpensive alternative medication that patients could take for VTE prophylaxis as the simple oral administration may improve the compliance rate. Haac et al. performed a randomised control trial looking at the post-discharge adherence of anticoagulation (Aspirin and LWMH). They found that although overall adherence was high, patients who took LMWH had a lower adherence compared to aspirin [17].

There were limitations to the study due to the retrospective nature. We were unable to assess patient-specific compliance to drug administration after discharge from the hospital. This is an important factor in determining the risk of VTE as adherence to regular medication administration can significantly increase the VTE risk. 

To our knowledge, this is the first study in the United Kingdom looking at adherence to the latest thromboprophylaxis guidelines in elective lower limb orthopaedic patients. This study allowed us to observe subsequent events once patients were discharged from the hospital following surgery. The two major strengths of this study are the large patient sample and long follow-up.

## Conclusions

VTE is a serious and potentially fatal complication that can occur in patients undergoing orthopaedic procedures. These patients should be administered VTE prophylaxis as an inpatient and on discharge if their weight-bearing status is affected. At our trust, a large series of 586 patients were reviewed, and our VTE incidence was 0.5%. Based on the review of our practice within one hospital trust, the NICE guidelines for reducing the risk of VTE in over 16s should be strictly adhered to as this will confer a lower rate of VTE complications as seen in our results. In order to further assess and manage the risks of lower limb surgery, further research should investigate the patient associated risks factors that can affect VTE and how this can be managed perioperatively. In addition, further work can take place determining the gold standard anticoagulant to use as there is no consensus within the literature. Variability in the type of anticoagulant prescribed in these patients may affect the VTE rates due to drug efficacy and patient compliance.
